# Predictive value of combining maternal peripheral blood count indicators for early-onset sepsis in preterm infants: A retrospective cohort study

**DOI:** 10.1097/MD.0000000000030526

**Published:** 2022-09-09

**Authors:** Yiwei Yan, Lian Jiang, Mei Li, Wenhao Zhang, Lingjuan Yu, Yuansu Zhang

**Affiliations:** a The Department of Pediatrics, The Fourth Hospital of Hebei Medical University, Shijiazhuang, PR China.

**Keywords:** early-onset sepsis, maternal neutrophil-to-lymphocyte ratio, mean platelet volume, nomogram, platelet to lymphocyte ratio

## Abstract

To assess the early predictive value of maternal parameters for early-onset sepsis (EOS) in preterm infants, especially including the maternal neutrophil-to-lymphocyte ratio (NLR), platelet-to-lymphocyte ratio (PLR), and mean platelet volume (MPV). The retrospective cohort study examined a total of 231 preterm infants (69 with EOS) from May 2017 to October 2021 of the Fourth Hospital of Hebei Medical University, randomly divided (7:3) into the training set group (n = 162) and validation set group (n = 69). Thirteen clinical variables (including MPV, NLR, and PLR) were included as the research objects. By logistic regression, the factors significantly associated with EOS were distinguished. Additionally, a nomogram was constructed based on the independent risk factors, the validation of which relied on the concordance index, calibration curves, receiver operating characteristic curves, and decision curve analyses. Multivariate logistic regression proved that NLR (OR = 1.67, 95% CI = 1.18–2.36, *P* = .004), PLR (OR = 1.03, 95% CI = 1.01–1.04, *P* = .001), and MPV (OR = 1.75, 95% CI = 1.15–2.66, *P* = .009) were independent risk factors for EOS. The AUC of the nomogram for the training set group was 0.872 (0.814, 0.931) and 0.889 (0.843, 0.935) in the validation set group. The *P* values of Hosmer–Lemeshow test for the training set and validation set groups were .903 and .752, respectively. The decision curve analyses outcome indicated good clinical practicability. The C-index for the training set and validation set groups were 0.872 and 0.889, respectively. The maternal NLR, PLR, and MPV levels had good predictive value for EOS in premature infants. The nomogram in our study could help clinicians predict the occurrence of EOS.

## 1. Introduction

The prognosis of preterm infants has attracted widespread attention. Among the conditions threatening the healthy survival of infants, neonatal sepsis has been recognized as a major and common complication. In the past 20 years, the global incidence of neonatal sepsis (among live births) was approximately 0.4%.^[1]^ The incidence of neonatal sepsis in China was 0.256%,^[2]^ and the mortality rate was more than 10%.^[3]^ Early-onset sepsis (EOS) refers to sepsis diagnosed within 72 hours after birth. EOS is closely related to maternal infection. It can be directly caused by maternal infection through the placenta or occurs as a retrograde infection induced by predisposing factors, such as vaginitis and premature rupture of membranes (PROM).

In preterm infants, the onset of EOS is usually insidious, and the lack of effective early predictive indicators may result in a delayed or missed diagnosis, thereby increasing the risk of poor prognosis. Although numerous preceding studies have explored the early predictive diagnosis of EOS, candidate indicators often lack specificity and clinical practical value. Previous studies mostly focused on the clinical manifestations and laboratory tests of newborns, ignoring the predictive value of maternal prenatal laboratory indicators and clinical status. In the 24 hours before delivery, pregnant women admitted to medical clinic services may undergo regular routine blood examinations; therefore, the neutrophil count, lymphocyte count, platelet count, and mean platelet volume (MPV) are easily obtained. According to recent studies, the neonatal neutrophil-to-lymphocyte ratio (NLR), platelet-to-lymphocyte ratio (PLR), and MPV had predictive value for EOS.^[4–7]^ Moreover, studies on adult sepsis reported that the values of NLR, PLR, and MPV could be clinically applicable indicators for predicting survival.^[8–10]^ Accordingly, it was suggested that the maternal NLR, PLR, and MPV within 24 hours before delivery could contribute to the prediction of EOS in premature infants. If this hypothesis was confirmed, it could shift the predicted time window to comply with the prenatal period, thereby improving the effectiveness of early prediction and diagnosis. To verify the predictive value of maternal diagnostic indicators, the laboratory indices and clinical features require in-depth assessments and validation based on patient information, thereby improving the accuracy of EOS diagnosis in clinical practice.

This study constructed a nomogram model based on EOS risk factors, which aimed to provide a reference for the early detection of EOS risk in preterm infants.

## 2. Materials and Methods

### 2.1. Patients and ethics

**Figure 1.**
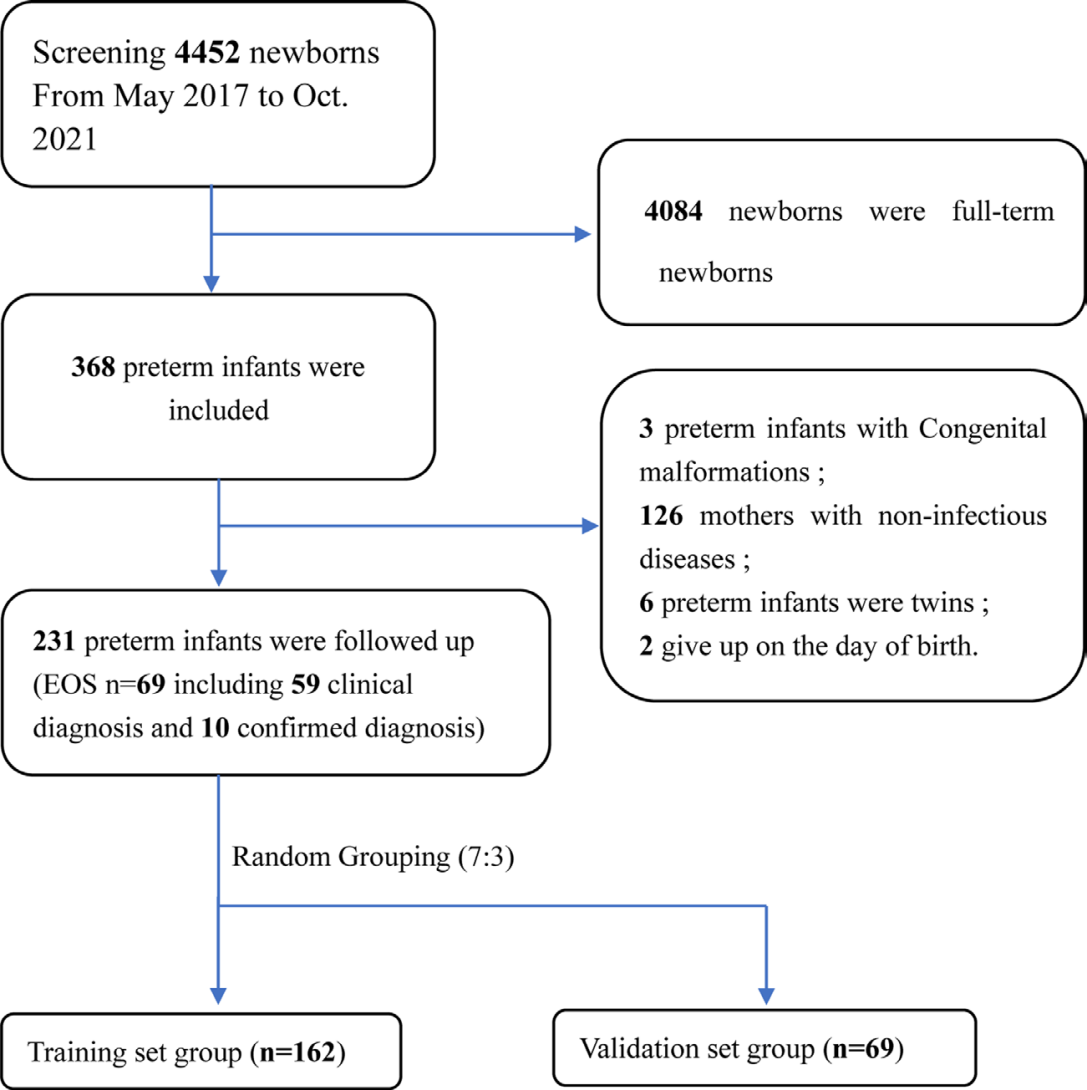
The research flow chart for this study. EOS = early-onset sepsis.

This was a single-center retrospective cohort study conducted from May 2017 to October 2021 (Total: 4452 cases). Infants born in the east district of the Fourth Hospital of Hebei Medical University were included for selection in our study. There were 368 cases of preterm infants among all newborns. The inclusion criteria were as follows: having complete clinical data in our hospital; the gestational age at birth was <37 weeks; all EOS patients met the EOS diagnostic criteria^[11]^ (detailed diagnostic criteria as below). Notably, preterm infants with any of these conditions were excluded: severe system malformations, inherited metabolic diseases, or severe perinatal asphyxia; pregnant mothers with noninfectious diseases before the delivery of preterm infants, such as lung diseases, endocrine, and metabolic diseases (such as diabetes and dyslipidemia), gestational hypertension, blood disorders, such as thrombocytopenia, eclampsia, tumors, liver and kidney diseases, anemia, toxic radiation exposure, smoking and alcohol exposure, heart diseases, cerebrovascular events, undergoing chemoradiation, etc; twin pregnancy or multiple pregnancy; a follow-up of <3 days. Consequently, 231 cases of preterm infants (male: 134 cases; female: 97 cases) of birth weight 520–3905 g were selected for the analyses in this study. In order to verify the accuracy of the model in a better manner, we randomly divided the included data into a training set group (n = 162) and a validation set group (n = 69) in a ratio of 7:3. The case registration process is shown in Figure 1.

The collection and publication of patient information had been approved by the Ethical Committee of the Fourth Hospital of Hebei Medical University (Hebei, Shijiazhuang, China; Ethical approval number: 2020004). This study deals with the field of pregnant women and newborns. This study does not disclose the data of the research participants in order to protect their personal information and respect their privacy. The content/data of this study may be obtained from the corresponding author upon reasonable request.

### 2.2. Clinical and laboratory variables

The following clinical variables were selected: sex of the newborn (male: 134; female: 97), birth weight (520–3905 g), gestational age (24–36^+6^ weeks; according to Corsi’ research^[12]^: <28 weeks for extremely preterm; 28–31^+6^ weeks for very preterm; 32–33^+6^ weeks for moderately preterm; 34–36^+6^ weeks for late preterm), the time of premature rupture of membranes (0–576 hours, *“*0 h*”* defined as “no premature rupture of membranes”), maternal body temperature 3 days before delivery (temperature < 38°C: 219 cases; temperature ≥ 38°C: 12 cases), mode of delivery (normal delivery: 58 cases; cesarean section: 173 cases), vaginitis (positive: 8 cases(Among positive cases, 3 cases were diagnosed with *Candida* vaginitis, 2 cases were of *Mycoplasma urealyticum* vaginitis, 1 case was gram-negative *Diplococcus* vaginitis, and 1 case was *Trichomonas* vaginitis); negative: 223 cases), and amniotic fluid pollution degree III (positive: 10 cases; negative: 221 cases; amniotic fluid pollution degree III^[13]^ defined as yellowish-brown, viscous amniotic fluid combined with yellowish fetal membranes, amniotic fluid contaminated with meconium).

The following laboratory variables were selected: maternal routine blood test results (samples taken within 24 hours before delivery), including white blood cell count, neutrophil count, lymphocyte count, platelet count, and mean platelet volume. NLR (neutrophil count/lymphocyte count) and PLR (platelet count/lymphocyte count) were calculated based on the results.

### 2.3. Follow-up

On the basis of consented acquirement of the data on hospitalization, preterm infants were followed up for 3 days; the number of cases diagnosed with EOS within 72 hours after birth were recorded (n = 69). A total of 162 subjects were not diagnosed with EOS.

To ensure the precision of EOS diagnosis for this study, the diagnostic criteria^[11]^ were specified as follows: Clinical Criteria for the Diagnosis of Sepsis: Neurologic: convulsions, drowsy or unconscious, decreased activity, bulging fontanel; Respiratory: respiratory rate > 60 breaths/min, grunting, severe chest indrawing, central cyanosis; Cardiac: poor perfusion, rapid and weak pulse; Gastrointestinal: jaundice, poor feeding, abdominal distention; Dermatologic: skin pustules, periumbilical erythema or purulence; Musculoskeletal: edema or erythema overlying bones or joints; Other: Temperature > 37.7°C (99.9°F; or feels hot) or < 35.5°C (95.9°F; or feels cold). Culture-Based Diagnostics (confirmed diagnosis): Positive blood culture (gold standard for bacteremia) or cerebrospinal fluid (CSF)-positive culture or positive endotracheal culture (in infants with an endotracheal tube). Positive urine culture does not prove to be useful for EOS. Non-culture-based Diagnostics (clinical diagnosis): Commonly used diagnostic tests include the total WBC (white blood cell) count and differential count and the ratio of immature to total neutrophils. A total neutrophil ratio of ≥ 0.2 suggests bacterial infection (neutropenia, neutrophilia, thrombocytopenia). The tests to demonstrate an inflammatory response include determination of C-reactive protein (CRP), procalcitonin (PCT), haptoglobin, fibrinogen, proteomic markers in amniotic fluid, inflammatory cytokines, and cell surface markers. Additionally, CSF-related tests also need to be considered: CSF WBC > 20 cells/mm^3^; CSF protein > 100 mg/dL; CSF glucose < 70% to 80% of serum glucose.

### 2.4. Statistical analysis

The SPSS 20.0 (Chicago, IL) and *R* software (version 3.5.3) were utilized for statistical calculations and graphing. There was no missing date. A logistic regression model was used to distinguish the risk factors of EOS. The least absolute shrinkage and selection operator (LASSO) regression was used to the effective variables. Based on the independent risk factors, a nomogram model was constructed with the *R* 3.5.3 program. Furthermore, the consistency index (C-index), calibration, and AUC (area under the ROC curve) were acquired to evaluate the performance. The sensitivity and specificity of the nomogram were determined by receiver operating characteristic (ROC) curves. In addition, the Hosmer–Lemeshow test was used to test the model fit. Calibration was achieved by plotting the correlation between the predicted probable outcomes and actual results in the form of a chart. A decision curve analysis (DCA) could present the significance value of the nomogram in a straightforward manner and verify the result. Additionally, the accuracy and consistency of the prediction model were verified through the validation set. Differences with *P* value < .05 were considered as statistically significant.

## 3. Results

### 3.1. Patient characteristics

Based on the selection criteria, a total of 231 preterm infants were included. Among them, 69 preterm infants were diagnosed with EOS, including 59 clinical diagnoses and 10 confirmed diagnoses. There were no obvious differences (*P > *.05) between the validation set and the training set groups among clinical characteristics, including sex, MPV, mode of delivery, fever, vaginitis, amniotic fluid degree III°, WBC, NLR, PLR, and PROM (Table 1).

**Table 1:** General characteristics of the study groups.

Variables	Training set group (n = 162)	Validation set group (n = 69)	*P* value
Sex, n (%)			
Male	99 (61.1)	35 (50.7)	.187
Female	63 (38.9)	34 (49.3)	
Birth weight (g)			
Median (IQR)	2020.0 (1538.0, 2458.0)	2045.0 (1451.0, 2600.0)	.906
Gestational age, n (%)			
<28 wk	14 (8.6)	6 (8.7)	.997
28–31^+6^ wk	39 (24.1)	16 (23.2)	
32–33^+6^ wk	22 (13.6)	10 (14.5)	
34–36^+6^ wk	87 (53.7)	37 (53.6)	
WBC (10^9^/L), median (IQR)	9.7 (8.0, 12.2)	9.0 (7.4, 11.3)	.282
NLR, Median (IQR)	4.6 (3.3, 6.9)	4.1 (2.8, 6.0)	.259
PLR, Median (IQR)	130.2 (100.8, 175.7)	136.5 (106.2, 171.4)	.758
MPV (fL), median (IQR)	10.1 (9.0, 11.2)	10.1 (9.3, 11.2)	.997
Mode of delivery n (%)			
Cesarean section	120 (74.1)	53 (76.8)	.785
Normal delivery	42 (25.9)	16 (23.2)	
P_pom_ (h), median (IQR)	0.5 (0.0, 2.0)	0.0 (0.0, 1.0)	.173
Fever, n (%)			
T < 38°C	155 (95.7)	64 (92.8)	.350
T ≥ 38°C	7 (4.3)	5 (7.2)	
Vaginitis, n (%)			
No	157 (96.9)	66 (95.7)	.699
Yes	5 (3.1)	3 (4.3)	
Amniotic fluid degree III°, n (%)			
No	153 (94.4)	68 (98.6)	.289
Yes	9 (5.6)	1 (1.4)	

IQR = interquartile range, MPV = mean platelet volume, NLR = neutrophil to lymphocyte ratio, PLR = platelet to lymphocyte ratio, P_pom_ = premature rupture of membranes, T = temperature, WBC = white blood cell count.

### 3.2. Risk factors for EOS

Univariate and multivariate logistic regression analyses were performed to distinguish the independent risk factors for EOS preterm infants. The univariate analysis showed that birth weight, gestational age, maternal WBC, NLR, and PLR were the candidate EOS predictors (*P < *.05). Furthermore, all the variables identified by the univariate analysis were used as independent variables, and a multivariate logistic regression analysis proved that NLR (OR = 1.67, 95% CI = 1.18–2.36, *P* = .004), PLR (OR = 1.03, 95% CI = 1.01–1.04, *P* = .001), and MPV (OR = 1.75, 95% CI = 1.15–2.66, *P* = .009) were independent risk factors for EOS. It should be noted that gestational ages were divided into 4 groups (OR = 0.18, 95% CI = 0.03–1.11 for 28–31^+6^ weeks, *P* = 065; OR = 0.04, 95% CI = 0–0.43 for 32–33^+6^ weeks, *P* = .007; OR = 0.01, 95% CI = 0–0.14 for 34–36^+6^ weeks, *P* = .001). In particular, there was no statistical difference in the group of 28–31^ + 6^ weeks. Owing to insufficient consistency in group prediction, our study did not include the gestational age as an independent risk factor (Table 2).

**Table 2:** Logistic regression analysis of the risk factors for EOS in preterm infants.

Variable	Univariate analysis		Multivariate analysis	
	OR (95% CI)	*P* value*	OR (95% CI)	*P* value^†^
Sex				
Male	Ref		Ref	
Female	1.53 (0.87–2.69)	.144	0.96 (0.34–2.68)	.937
Birth weight (g)	1 (1–1)	<.001	1 (1–1)	.711
Gestational age, (%)				
<28 wk	Ref		Ref	
28–31^+6^ wk	0.3 (0.09–1.01)	.053	0.18 (0.03–1.11)	.065
32–33^+6^ wk	0.13 (0.04–0.49)	.002	0.04 (0–0.43)	.007
34–36^+6^ wk	0.03 (0.01–0.09)	<.001	0.01 (0–0.14)	.001
WBC (10^9^/L)	1.27 (1.16–1.4)	<.001	0.98 (0.78–1.22)	.837
NLR	1.77 (1.5–2.09)	<.001	1.67 (1.18–2.36)	.004
PLR	1.03 (1.02–1.04)	<.001	1.03 (1.01–1.04)	.001
MPV (fL)	0.88 (0.74–1.05)	.155	1.75 (1.15–2.66)	.009
Mode of delivery				
Cesarean section	Ref		Ref	
Normal delivery	0.96 (0.5–1.85)	.914	1.47 (0.44–4.88)	.529
P_pom_ (h)	1 (1–1.01)	.168	1 (0.99–1.01)	.535
Fever, n (%)				
T < 38°C	Ref		Ref	
T ≥ 38°C	1.73 (0.53–5.65)	.364	5.11 (0.82–31.91)	.081
Vaginitis, n (%)				
No	Ref		Ref	
Yes	2.43 (0.59–10.01)	.219	5.73 (0.79–41.67)	.085
Amniotic fluid degree III°, n (%)				
No	Ref		Ref	
Yes	1.6 (0.44–5.86)	.478	1.13 (0.07–17.6)	.931

CI = confidence interval, MPV = mean platelet volume, NLR = neutrophil to lymphocyte ratio, OR = odds ratio, PLR = platelet to lymphocyte ratio, P_pom_ = premature rupture of membranes, Ref = reference, T = temperature, WBC = white blood cell count.

* *P* for the result of univariate analysis.

† *P* for the result of multivariate analysis.

### 3.3. Construction and validation of nomogram to predict EOS probability

**Figure 2.**
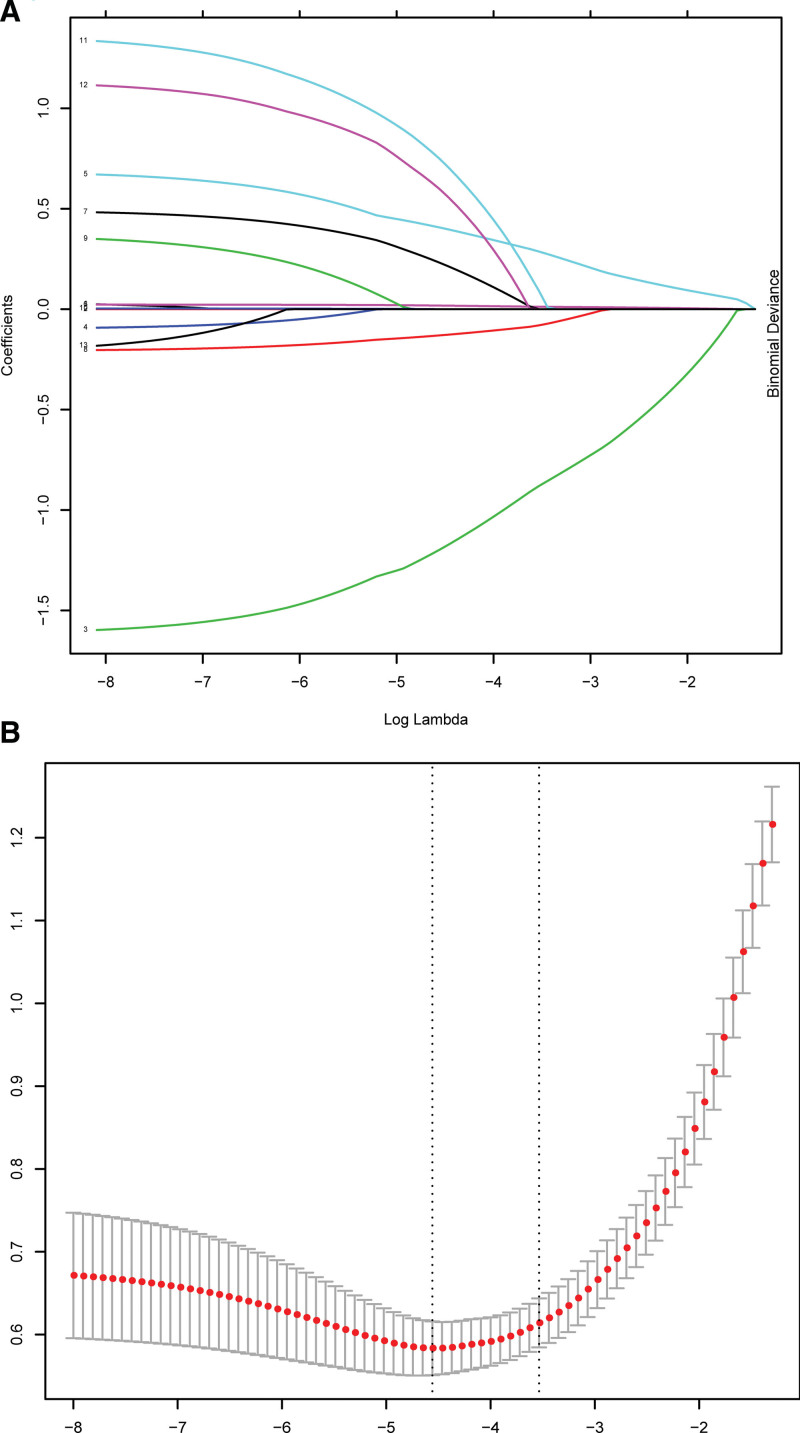
Variables selection using LASSO and multivariate Logistics regression analysis. (A (left), B (right)) Selection of the most appropriate penalty parameter (λ) for LASSO regression, and the LASSO regression selected 4 variables. LASSO = least absolute shrinkage and selection operator.

According to multivariate logistic regression, NLR, PLR, and MPV were independent risk factors for positive EOS. LASSO regression was a shrinkage method, reducing the likelihood of overfitting by actively selecting from a large set of variables and cutting down the regression coefficient.^[14]^ LASSO regression tests were conducted on all variables in the study, and the selected variables were consistent with independent risk factors (Table 3, Fig. 2).

**Table 3:** Selected variables of LASSO regression analysis.

Variable	Estimate	SE	*Z* value	*P* value
Gestational age, (%)				
32–33^+6^ wk	−3.159	0.97	−3.257	0.001
34–36^+6^ wk	−4.906	1.011	−4.853	0
NLR	0.493	0.127	3.877	0
PLR	0.027	0.007	4.061	0
MPV	0.547	0.202	2.707	0.007

LASSO = least absolute shrinkage and selection operator, MPV = mean platelet volume, NLR = neutrophil to lymphocyte ratio, PLR = platelet to lymphocyte ratio, SE = standard error.

**Figure 3.**
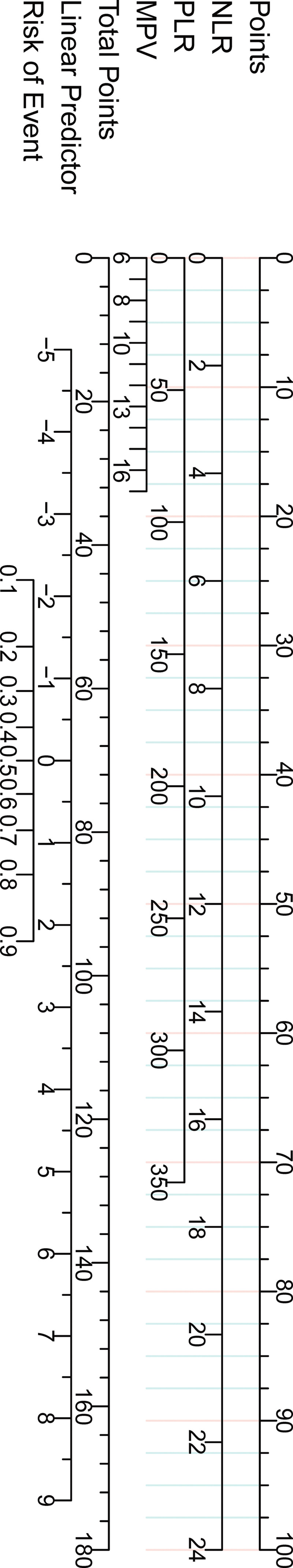
Nomogram model for predicting the incidence of EOS in preterm infants. EOS = early-onset sepsis, MPV = mean platelet volume, NLR = neutrophil to lymphocyte ratio, PLR = platelet to lymphocyte ratio.

**Figure 4.**
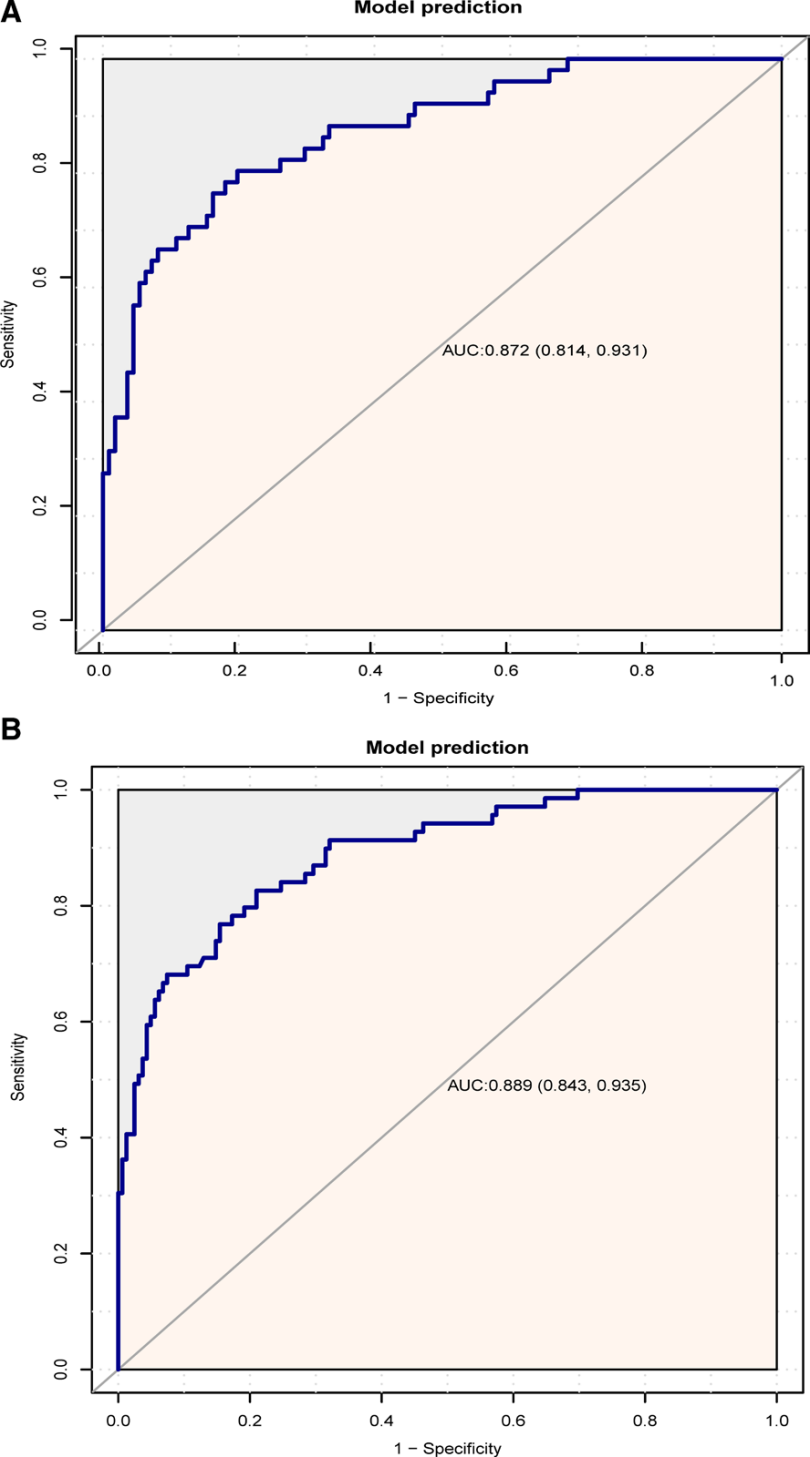
ROC curve for evaluating the reliability of the prediction model. A(left) for training set group; B(right) for validation set group. ROC = receiver operating characteristic.

**Figure 5.**
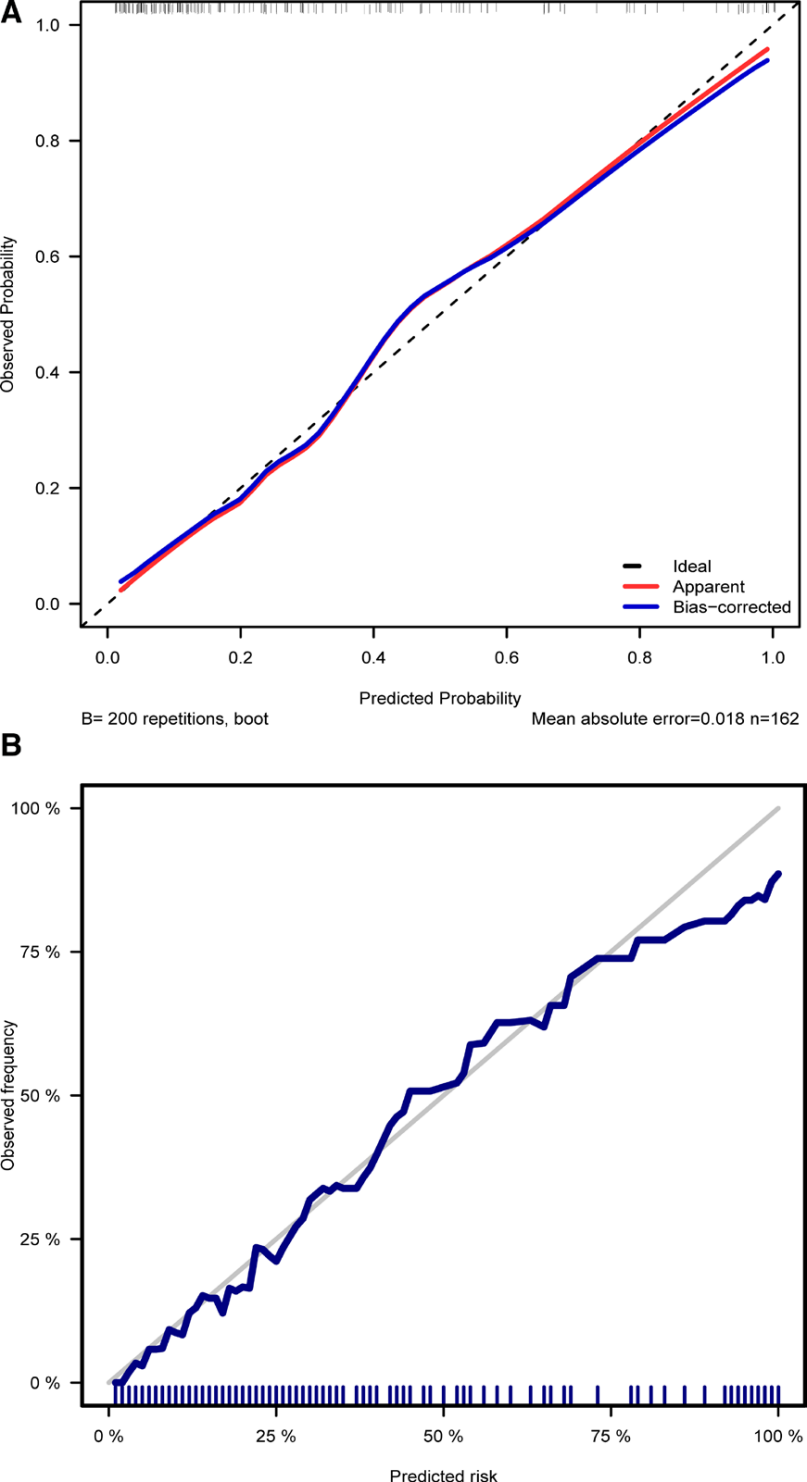
Calibration curves for EOS incidence prediction. A(left) for training set group; B(right) for validation set group. EOS = early-onset sepsis.

**Figure 6.**
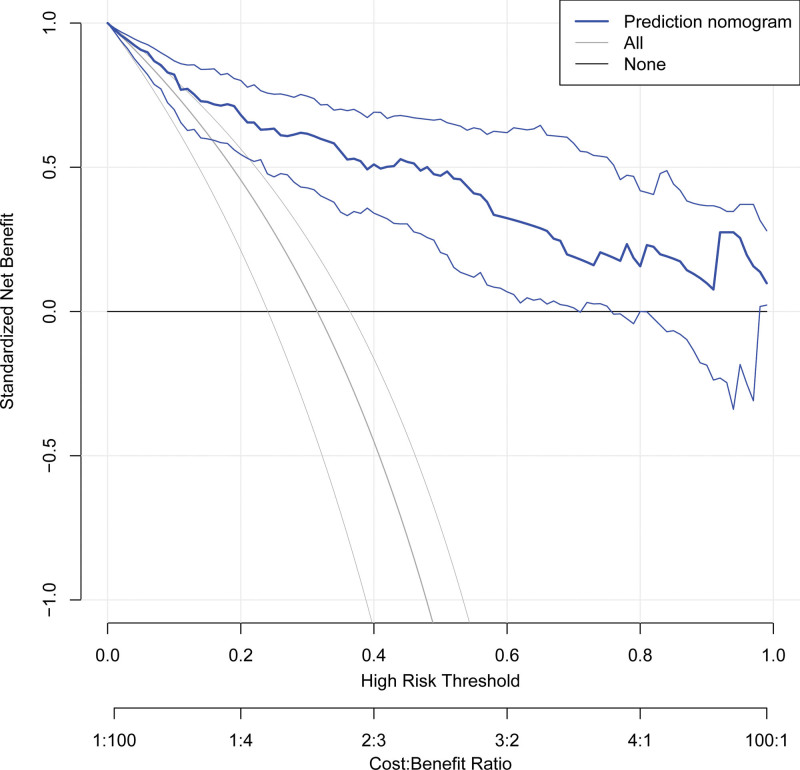
The results of the decision curve analysis (DCA) for nomogram.

Based on these independent risk factors (NLR, PLR, and MPV), a nomogram was constructed to predict EOS in preterm infants (Fig. 3). The AUC of the nomogram for the training set group (Fig. 4A) was 0.872 (0.814, 0.931), and the AUC for the validation set group (Fig. 4B) was 0.889 (0.843, 0.935). The *P* values of Hosmer–Lemeshow test for the training set and validation set groups were .903 and .752 (*P* > .05), respectively, indicating that the difference between the predicted value and the real value was insignificant. These results suggested a good model fit. The calibration curve showed a favorable prediction accuracy of the nomogram (Fig. 5A for the training set group; Fig. 5B for the validation set group). The DCA outcome of the training set group indicated good clinical practicability (Fig. 6). The C-index for training set and validation set groups were 0.872 and 0.889, respectively.

## 4. Discussion

Although the correlation between complete blood count and EOS risk has been examined in clinical studies, most of them focused on term infants. However, the incidence and severity of EOS are more pronounced in premature infants. In the United States, the incidence of sepsis in preterm infants has approximately tripled compared to that in term infants.^[15]^ Recently, numerous early predictive indicators for EOS have been suggested; however, their clinical practicability still required further validation in large-scale studies. Therefore, although the incidence and flora of EOS have changed, most of the predictive and diagnostic indicators conventionally used in clinical practice have not been replaced by novel candidate indicators, and the accuracy of diagnosis has not been satisfactorily improved. According to multiple previous studies, maternal diagnostic indicators should be combined with the neonatal clinical parameters in a specific manner so as to predict and evaluate the occurrence and development of EOS in a more strategic way, thereby enabling earlier and effective administration of treatment, as well as reducing the application of antibiotics.^[15,16]^ Considering the current clinical demand, our study selected preterm infants as the research objects, which were stratified according to the gestational ages. Overall, 12 maternal-related clinical factors were evaluated with statistical approaches to distinguish independent risk factors and to construct a predictive model.

Since performing a complete blood count test is fast, convenient, and simple, it is expected that a blood test can be used to predict the occurrence of EOS, thereby remarkably reducing the cost of disease monitoring and diagnosis. In our current study, as proved by univariate analyses, birth weight, gestational age, maternal WBC, NLR, and PLR were the candidate predictors for EOS. In order to further exclude the influence of interfering factors, a multivariate analysis was performed, and the results suggested that maternal NLR, PLR, and MPV were independent risk factors. In recent years, several research studies have examined the relationship between individual items of complete blood count and sepsis. Can et al carried out a study on EOS in term infants and found that the levels of neonatal NLR and PLR with positive EOS were significantly elevated compared with those indices of the non-EOS group. Moreover, the cutoff values of NLR and PLR obtained from the ROC curve were observed to be 6.76 and 97.4, respectively.^[4]^ In 2019, a meta-analysis of sepsis including 14 studies (more than 10,000 patients) indicated that NLR was not only related to the occurrence of infection, but also significantly increased in deaths from sepsis, suggesting poor prognosis.^[17]^ Through a retrospective cohort study, Arcagok et al^[5]^ found that the peripheral blood PLR of neonates diagnosed with EOS or suspected with EOS showed a remarkable increase compared to that of non-EOS neonates. Furthermore, the AUC of PLR was approximately 90%, which could be used to predict the occurrence of EOS. In addition, in patients with sepsis induced by peritonitis, MPV was recognized as an independent risk factor and closely related to poor prognosis.^[10]^ Moreover, according to a meta-analysis on neonatal sepsis, for the neonatal sepsis group in the overall analysis and subgroup analysis, the increase in MPV was significant, suggesting the potential of MPV as an indicator for the early diagnosis of neonatal sepsis in clinical practice.^[8]^ These research results were highly consistent with the conclusions of our article.

However, most previous studies on the diagnosis of neonatal sepsis focused on the indicative factors of the infants. Owing to the physiological background of the major conventional indicators for neonatal sepsis in premature infants, the cut-off value of CRP, PCT, and blood routine required for EOS diagnosis could be affected by the postnatal time (such as 6, 24, and 72 hours). Furthermore, blood count indicators of newborns are susceptible to environmental factors, thereby limiting the diagnostic specificity and the effectiveness of early prediction. On the contrary, adult blood indicators are relatively stable, with a minor chance of significant changes induced by external factors. For the candidate EOS predictive indicators, the maternal parameters were selected among the peripheral blood count within 24 hours before delivery. They could not only reflect the prenatal infection status of the mother, but also shift the predicted time window to comply with the prenatal period, thereby improving the effectiveness of early prediction and diagnosis. Furthermore, compared with the statistical methods applied in the above literature, our study can more accurately predict the occurrence of EOS by constructing a nomogram.

Neonatal CRP and PCT have been assessed in-depth as EOS diagnostic indicators. In a previous study, the sensitivity of CRP and PCT were <0.75.^[18]^ In another study, the AUC value of the combined diagnosis of PCT and IL-6 was 0.801, and the sensitivity was 0.88, while the AUC value of PCT and CRP combined diagnosis was 0.819, and the sensitivity was 0.82.^[19]^ In our study, the ROC curve was generated to determine the AUC value for maternal NLR, PLR, and MPV in combination. The results were satisfactory to prove the clinical potential. The AUC values of the indicators suggested in our study were higher than those of the above studies, including the AUC value of neonatal PCT and CRP combined diagnosis. The sensitivities had little differences compared with the results of the above studies. These statistical results confirmed the predictive value of NLR, PLR, and MPV for EOS in preterm infants, thereby verifying the clinical feasibility of our study to a certain degree.

Prematurity is an important basis of disease for the occurrence of EOS. Despite these independent risk factors passing statistical validation, gestational age particularly showed extra complexity in the statistical analyses to prove the predictive significance. Data from a multi-center study (18 perinatal care centers) showed that preterm infants with a gestational age of <34 weeks had an EOS incidence of 0.97% and case fatality rate of 22%.^[20]^ A shorter gestational age could be associated with substantially greater disease burden. In very premature infants, as well as with very low birth weight infants, the morbidity and mortality of EOS could be 3 to 4 times of those in late preterm infants.^[21]^ Several studies confirmed that the gestational age was closely related to the occurrence of EOS. According to the gestational age stratification of preterm infants included in this project, the incidences of EOS in ultra-premature infants, very preterm infants, mid-term preterm infants, and late preterm infants were 80%, 55%, 30%, and 9.8%, respectively. This finding was in line with the conclusions of the above studies, supporting the relationship between the incidence of EOS in preterm infants with the gestational age. In this study, the gestational ages were divided into 4 classes. As per the results of multi-factor analysis, one of the classes (28–31^ + 6^ weeks) was statistically significant. For rigorous consideration of validity, gestational age could not be considered to be an independent risk factor in our study; however, the impact of gestational age on EOS was still noticeable. Moreover, Mei et al^[22]^ proposed the use of maternal CBC and its derived parameters to predict the occurrence of asymptomatic preterm birth. They recognized the parameters as inflammatory markers for low-grade inflammatory diseases. However, this article does not further explore the correlation with infectious diseases in preterm infants. Preterm birth is an important basis of disease for the occurrence of EOS; nevertheless, there are several other factors that can affect the occurrence of EOS. Therefore, the occurrence of EOS cannot be judged by predicting preterm birth accurately. The target disease of our research is EOS; the occurrence of EOS is predicted by detecting maternal CBC, and the possibility of EOS occurrence is more accurately predicted by constructing a nomogram.

At present, few studies have used maternal complete blood counts to establish nomograms for predicting EOS in preterm infants. Compared with other mathematical models, the nomogram displays the contribution rate of each risk indicator as the length of the line segment, which is straightforward and informative. Based on the ease of interpretation that resulted from visualization, complex algebraic calculations can be saved, and the verification of clinical practicability can be efficiently facilitated. In our study, the nomogram model was constructed based on three independent risk factors, the selection of which relied on logistic regression and LASSO regression methods. To avoid over-fitting of the model, internal and external data were introduced to conduct multiple verification methods. The C-index of the training set group and the validation set group were satisfied. The calibration curve fitted well with the ideal curve, and the AUC for the training set group, as shown in Figure 4A, and the AUC of the nomogram for the validation set group, as shown in Figure 4B, were more systematic than the verification with a single ROC curve. According to the nomogram, the occurrence of EOS could be predicted based on the sum of the scores of individual risk factors, thereby improving the screening of EOS risk and the management of controllable factors. Our nomogram could also remind clinical staff to recognize the clinical significance of maternal risk factors in the diagnosis of EOS for preterm infants. In clinical daily practice, some manners could be taken to manage high-risk preterm infants. Such as regular monitoring of infection indicators, shortening the interval between reexamination, and closely observation of infection-related clinical signs. Accordingly, the prediction and diagnosis of EOS might be achieved in a timely manner to facilitate the application of appropriate and effective therapies, thereby cutting down the mortality of EOS preterm infants, decreasing the use of antibiotics, reducing the development of drug resistance, avoiding multiple organ damage caused by infection, and improving the prognosis.

## 5. Limitations of the study

First, the retrospective study, which selected patients according to specified criteria, had a common intrinsic shortage. Compared with prospective studies, the possibilities of including inaccurate data and the limited selection range were inevitable. Therefore, we did not include all the possible variables as confounding factors in the study. Our principle was to first recognize the influencing factors, and then to observe what can be obtained from the cases. Therefore, the use of antibiotics, urban or rural areas, and educational background were not included in the study. Because the data of the above factors are small, they cannot be correctly counted, and there is no clear evidence to prove that they are important confounding factors. This may be a limitation; however, it is also an established fact of retrospective studies. Accordingly, large-scale prospective clinical studies are required to further verify the conclusion. Subsequently, although internal verification was satisfactory to validate our model, external validation was still necessary for the evaluation of clinical potency. In the next step, we will further cooperate with the pediatric departments of multiple hospitals to carry out multi-center research to verify the accuracy of the model and further build a more complete evaluation system. Thirdly, owing to the current routine protocol of early treatment, numerous preterm women had not been tested for infection indicators, such as CRP and PCT before delivery; therefore, such potential indicators could not be included in this study. Finally, the current scale of clinical data was inadequate, and therefore the credibility of the results could be enhanced by enlarging the scale of the follow-up study in future.

## 6. Conclusion

By using retrospective cohort studies, we found that maternal NLR, PLR, and MPV levels (within 24 hours before parturition) had a good predictive value for EOS in premature infants. The nomogram in our study could help clinicians better predict the occurrence of EOS; additionally, it may contribute to the prevention and the management of EOS in preterm infants.

## Acknowledgments

The authors acknowledge the efforts of Dr Xuhua Hu’s efforts in data statistics. The above doctor did not have any writing assistance.

## Author contributions

**Conceptualization:** Yiwei Yan, Lian Jiang.

Data curation: Mei Li, Yuansu Zhang, Lingjuan Yu, Wenhao Zhang.

Formal analysis: Lian Jiang, Yiwei Yan.

Investigation: Yiwei Yan, Wenhao Zhang.

Project administration: Yiwei Yan.

Supervision: Lian Jiang, Yiwei Yan.

Validation: Lian Jiang, Yiwei Yan.

Writing – original draft: Yiwei Yan.

Writing – review & editing: Yiwei Yan.
